# Children with albinism in African regions: their rights to ‘being’ and ‘doing’

**DOI:** 10.1186/s12914-018-0144-8

**Published:** 2018-01-12

**Authors:** Anita Franklin, Patricia Lund, Caroline Bradbury-Jones, Julie Taylor

**Affiliations:** 1 Faculty of Health and Life Sciences, Coventry University, Priory Street, Coventry, CV1 5FB England; 2 Institute of Clinical Sciences, University of Birmingham Edgbaston, Birmingham, B15 2TT England

**Keywords:** Children with albinism, People with albinism, Barriers, Children’s rights, Disability, Human rights, Africa

## Abstract

**Background:**

Albinism is an inherited condition with a relatively high prevalence in populations throughout sub-Saharan Africa. People with oculocutaneous albinism have little or no pigment in their hair, skin and eyes; thus they are visually impaired and extremely sensitive to the damaging effect of the sun on their skin. Aside from the health implications of oculocutaneous albinism, there are also significant sociocultural risks. The impacts of albinism are particularly serious in areas that associate albinism with legend and folklore, leading to stigmatisation and discrimination. In regions of Africa those with albinism may be assaulted and sometimes killed for their body parts for use in witchcraft-related rites or to make ‘lucky’ charms. There is a dearth of research on the psychosocial aspects of albinism and particularly on how albinism impacts on the everyday lives of people with albinism.

**Discussion:**

There is a growing recognition and acceptance in Africa that people with albinism should be considered disabled. Thomas’s social-relational model of disability proposes it is essential to understand both the socio-structural barriers and restrictions that exclude disabled people (barriers to doing); and the social processes and practices which can negatively affect their psycho-emotional wellbeing (barriers to being). In this article, we combine a social model of disability with discussion on human rights to address the lacuna surrounding the psychosocial and daily experiences of people with albinism.

**Conclusion:**

Through using this combined framework we conclude that the rights of people with albinism in some regions of Africa are not being enacted. Our debate highlights the need to develop a holistic concept of rights for children and young people with albinism which sees human rights as indivisible. We illuminate some of the specific ways in which the lives of children with albinism could be improved by addressing ‘barriers to being’ and ‘barriers to doing’, at the heart of which requires a shift in attitude and action to address discrimination.

## Background

To date there is a paucity of research on the psychosocial aspects of albinism [1] and on the experiences of people with albinism (PWA). Significantly, the experiences and perspectives of children with albinism are underexplored and little is known about the psychosocial issues facing this group. Limited understanding of their lives in an African context and a lack of attention given to the particular needs of this group of children means that interventions to better support empower and protect them remain elusive.

Inspired by Thomas’ notions of ‘barriers to being’ and ‘barriergs to doing’ [2] and underpinned by a human rights approach, our innovative lens seeks to explore the challenges that PWA and their families face in some regions of sub-Saharan Africa, especially in countries where discrimination against PWA is common. Our integrated framework incorporates sensitive approaches to social protection, useful in countries of low economic resource [3]. It can keep us alert to the barriers ‘to being’ and ‘to doing’ that might exacerbate the vulnerability of PWA.

### Oculocutaneous albinism

Albinism is a genetic condition caused by a deficit in production of the pigment melanin. It affects populations across the globe regardless of gender or ethnicity although frequencies vary. There are different types of albinism, but the most common and visible is oculocutaneous albinism. There are few detailed epidemiological studies, but the prevalence of albinism has been approximated at around 1 in 2000–5000 throughout sub-Saharan Africa [4, 5]. Data on the prevalence of albinism amongst school children in Zimbabwe and South Africa estimates a prevalence rate in the region of 1 in 2000 to 4000 [6, 7]. Demographic data from 2011 in Namibia revealed the highest national prevalence reported in Africa to date at 1 in 1755, compared with 1 in 2673 in the 2012 census in Tanzania [8].

Children in African communities born with oculocutaneous albinism lack pigment in their skin, hair and eyes due to inherited recessive mutations in one gene. This has serious implications for their physical health and wellbeing. Lack of skin pigmentation means that they are very sensitive to the damaging effects of the sun. Harsh sunlight causes skin cancers and exacerbates eye conditions [9].

Albinism results in poor vision, with involuntary nystagmus, photophobia, poor depth perception, strabismus, poor visual acuity and refractive errors [7]. In one study 85% of children with albinism in South Africa were found to have less than 30% vision, even with best optical correction [8]. If not understood or managed incorrectly, albinism can have deleterious health and social impacts on the lives of children with the condition.

Aside from the physical consequences of oculocutaneous albinism, there are also significant sociocultural risks. In Africa the lack of pigmentation makes the visible appearance of those with the condition markedly different to their dark skinned families and communities. The impacts of albinism are particularly acute in regions of the world where myths and superstitions surrounding the condition can lead to stigmatisation, discrimination and additional health issues such as cancers due to a lack of adequate sun protection and appropriate treatment of early pre-cancerous lesions. In the last 10 years there have been cases of violent assault and murder as PWA are targeted for their body parts for use in witchcraft-related rituals to make charms believed erroneously to bring easy wealth and good fortune [10].

## Discussion

### Human rights and disability

Disability specifically refers to negative interactions between people with impairments and the internal and external environment [11]:

Disability is the umbrella term for impairments, activity limitations and participation restrictions, referring to the negative aspects of the interaction between an individual (with a health condition) and that individual’s contextual factors (environmental and personal factors).

There is a growing recognition and acceptance in Africa that PWA should be recognised as disabled [12], although on an individual level people may not necessarily accept or adopt such a label. In a recent study assessing prison life of disabled people in Ghana, albinism was named specifically as one of six categories of disability [13]. As disabled people, PWA are afforded human rights as set out by the United Nations Convention on the Rights of Persons with Disabilities (UNCRPD) [14]. These rights include a right to life, adequate standards of living and social protection, equality and non-discrimination, freedom from exploitation, violence and abuse, and a right to education, health, work and employment.

For a number of years the United Nations (UN) has underlined the extreme discrimination faced by people with albinism. In 2013 the UN Human Rights Council called for the prevention of attacks and discrimination against PWA [10] and in December 2014, the UN General Assembly adopted a resolution proclaiming the 13th of June each year as International Albinism Awareness Day [15]. Moreover, in March 2015 the Council created the mandate of Independent Expert on the enjoyment of human rights by PWA in response to groups advocating for PWA to be seen as a specific group with particular needs requiring attention [16]. More recently (2017) the International Bar Association proposed universal standards for the protection and promotion of rights for people with albinism [17].

### The children’s rights agenda

Within the African context children with albinism not only have rights as disabled people under the UNCRPD; they are also afforded rights under the United Nations Convention on the Rights of the Child (UNCRC) [18] which has been signed and ratified across Africa. Additionally, the 1999 African Charter on the Rights and Welfare of the Child was created to provide additional rights to protection and outlines the rights that African countries must ensure for their child population [19]. It is the main instrument of the African human rights system for promoting and protecting child rights, complementing the UNCRC in addressing issues of discrimination, empowerment and protection. Despite such legislative protection of rights, it is only recently that a detailed examination of the rights of children with albinism has been undertaken [17].

### Albinism: impairment effects and disablism

The social model of disability makes a distinction between *impairment*, the lost or limited functioning experienced by an individual, and *disability*, the barriers that people with impairments face because of the way societies are structured [20]. Barriers may be social, cultural, material, physical or attitudinal and they exclude people with impairments from mainstream life. As the evidence illustrates these barriers are frequent aspects of the everyday lives of PWA in Africa.

In the further development of the social model of disability, Thomas’ work on social relational understandings of disability also recognises the significance of *impairment effects*, meaning the day-to-day impact of living with particular conditions or impairments [2]. In the case of children with albinism, impairment effects include poor vision and potential skin damage due to the effects of the sun. Thomas argues that people may use such impairment effects to exploit a person, an act of disablism. Thomas argued for a two-pronged approach to understanding and tackling disablism: understanding the socio-structural barriers and restrictions that exclude disabled people (‘barriers to doing’); and understanding the social processes and practices which can negatively affect the psycho-emotional wellbeing of people with impairments (‘barriers to being’). This model has been used with effect in the study of disabled children [21].

Although social models of disability are predominately used in Western definitions of disability, Brocco argues that the definition of being a disabled person may constitute a way for PWA to find alternative and more suitable epistemologies for albinism than some of the dominant negative discourses [22]. He argues that adopting a disability definition may help PWA explain to others why they cannot do certain activities such as working outdoors – ‘impairment effects’. In our view, the most important contribution is that it gives PWA the same recognition for financial or other support afforded other disabled people.

### ‘Barriers to being’

There is evidence to show that social processes and practices severely affect children with albinism and as such they face significant ‘barriers to being’, most seriously, their right to life and protection and the right to freedom from discrimination.

### The right to protection

Although there are laws in Africa and beyond protecting PWA, these need to be implemented effectively [23]. International responses are criticised for being limited to little more than political rhetoric and only the media and NGOs are thought to have had any traction in raising awareness and conducting advocacy work [24]. In a recent study in the lake region of Tanzania respondents did praise the government’s awareness campaigns though, which are viewed as the main reason for an improvement in attitudes towards PWA (personal communication Lund). In the 2 years following 2008 when the Tanzanian government declared it a capital crime to kill people with albinism, more than 170 people were arrested in that country. However very few were prosecuted, with courts citing a lack of funds for litigation as the reason [25]. The situation seems little changed since that study [17].

In response to the extreme violence against children with albinism, the Tanzanian government implemented a policy of moving children with albinism from their family homes into special schools and camps in order to protect them [24]. It is argued that while emergency response might have increased security, concerns were raised about overcrowding, inadequate facilities and support, incidents of child abuse and family members abandoning their children in the facilities [24]. The impact of this form of segregation on family life has received less attention and even less so the impact on the wellbeing of the children.

Alongside a right to protection of life, PWA also have the right to health protection, including access to skin and eye health services and provision of skin protection materials such as protective clothing and sunscreens. The high risk of developing skin cancer makes it essential that children with albinism are taught about and are enabled to implement effective sun protection from an early age. However, in Lund’s study in Zimbabwe [26], only 23% of her sample of children had ever had their skin examined by a health professional and this was often only when they were babies. In terms of other health needs, less than half in this sample (46%) had been to a hospital or private optician for an eye test and only 27% had prescription glasses, 5% of who felt that they did not improve their eyesight. Similarly, in a sample of 38 children in a rural special school for visually impaired children in South Africa the ‘sun protection’ used by the children was often in the form of inexpensive aqueous creams with no sun protection [7]. Even where these did provide some protection it was of an inadequately low SPF value for oculocutaneous albinism [7]. Lund reports on the persistent skin and eye problems of children with albinism in a study in Zimbabwe, highlighting the lack of suitable health care facilities and social care support, with many families relying solely on support from within their families [27].

It is important to note that negative attitudes towards PWA are not unique to Africa. For example, Wan [28] reports on the disablism experienced by PWA predominately in North America and their strategies to resistance. Moreover attitudes on the African continent vary significantly and appear slightly more positive in West Africa in that it is not as threatening for PWA living there as in parts of Southern Africa (personal communication PL and J Epelle (CEO) The Albino Association of Nigeria and UN Albinism Champion).

### The right to live free from discrimination

Myths and superstitions, fuelled by a lack of understanding surrounding albinism and the visible difference in the appearance of PWA can lead to stigmatisation, rejection, a lack of acceptance, perceptions of difference and limited social integration [1, 22, 25]. Their visible difference is so stark that they are in effect viewed as white people within a black community. Bucaro [25] points out some of the cultural superstitions in Tanzania whereby the health issues faced by PWA are seen to be a result of a curse, or they are seen as ‘omens of disaster’. Furthermore Brocco [22] highlights the negative labels and terminology used to describe and define PWA in Africa. Mothers of children with albinism are often blamed for their child’s condition, accused of infidelity with white people, carriers or spirits [29]. Thus many children with albinism are raised without the support of both parents, creating financial difficulties and an inability to afford health care or education for their child [25]. The efforts of many NGOs in Africa aim to offer this support.

Other authors have explored the implications of both old and new myths about albinism on personal identity and social acceptance [29]. Over half of the pupils in a sample of schoolchildren in Zimbabwe did not know why their skin was pale – other common misconceptions being that they thought they had the top (black) layer of skin missing [27]. If well informed, teachers can be an important, effective route for dissemination of information about genetics and health care, especially as they are such highly respected members of the community [27].

Recent anthropological research has critiqued the media for their simplistic approach of blaming traditional superstitions for the killings of PWA, identifying that contract killers are fulfilling a market demand for body parts for use as charms thought to bring wealth and good fortune [24]. There are strong arguments that it is poverty that drives the violence towards PWA [25] and this is specifically acknowledged by the United Nations as a key contributor to witchcraft related violence [26]. It is possible to make a direct link to the boom in mining and rapid social change and inequalities in Tanzanian society [24, 30, 31] and though poverty in and of itself does not cause violence; in terms of PWA it seems to be a significant risk factor.

Media reports of violence against people with albinism have mostly focused on identifying and punishing the perpetrators, although there are articles focused on awareness raising and political activism [24]. Burke et al.’s content analysis of media reports illuminate how some of the strategies aiming to prevent abuse or violence are criticised and debated, highlighting the complexities involved in protecting and promoting competing rights [24].

The profound effects of this violence, on PWA as well as on the wider community, are also documented regularly in the media, illustrating how family members are forced to escort their children to school and to other areas of the community. There are accounts of parents hiding their children at home, seeking asylum in police stations or moving to safer parts of the country. Some families report sending their children away to boarding schools, camps or relations in safer areas [24]. Such media attention has also raised awareness of the need to address the challenges facing PWA, including stigma and the lack of access to education and health services [24].

Thomas emphasizes the importance of understanding the psychosocial effects of disablism [2]. Although there is a paucity of evidence on this aspect, a qualitative study conducted with 15 adults with albinism in South Africa highlighted the negative effects the disablist external environment can have on self-image and on their sense of belonging at home and within the wider community [1]. Previous studies have also identified that prejudice and stigma are a major challenge faced by most PWA [32, 33]. A sample of children with albinism in Zimbabwe reported problems they encountered around children avoiding, antagonising and indeed fearing them [28]. Further, the development of self-esteem through group and team activities taking place in outside spaces is being denied to this group [7].

Individual testimonials provide first-hand accounts of these difficulties. The first Member of Parliament with albinism to be appointed in Tanzania, Ms. Kway-Geer, described her life as a schoolchild:

*When I was at primary school, people used to laugh at me, tease me - some didn’t even like to touch me, saying that if they touched me they would get this colour. People used to abuse me on the road when I took the buses to school. They would run after me - crowds of kids following me - shouting ‘zeru, zeru’* (derogatory term for someone with albinism) [34].

Both children’s rights and those of disabled people are compromised by the ‘barriers to being’ faced by children and adults with albinism, summarised in Table 1.

**Table 1:** Evidence of Barriers to Being & the Unenacted Rights of People with Albinism (relevant Article in parentheses)

*Barriers to being*	*Rights afforded under the UNCRC (1989)*	*Rights afforded under the UNCRPD (2006)*
Exposure to/fear of violent assault, abduction & murder	➢ Right to life, survive & develop (6) ➢ Protection from violence, abuse & neglect (19). ➢ Governments must protect children from being abducted, sold or moved illegally for the purpose of exploitation (35). ➢ Children who have experienced abuses must receive support to help them recover (39).	➢ Right to live (10). ➢ Governments must take all necessary measures to ensure the protection & safety of people with disabilities in situations of risk (11). ➢ Right to freedom from exploitation, violence & abuse (16).
Effects of discrimination & stigmatisation	➢ Right to live full & decent lives with dignity & as far as possible, independence & to play an active role in their community (23).	➢ Right to equality & non-discrimination & equal protection & benefit of the law (5). ➢ Governments must raise awareness of the capabilities of disabled people & challenge discrimination & prejudice (8). ➢ Right to live independently in & be included in the community 19).
Effects of segregation from family &/or community & lack of freedom of movement	➢ If a child is place away from home for the purposes of protection they have the right to regular review of their care & circumstances (25).	➢ Right to access all aspects of society on an equal basis (9). Right to liberty & security of person (14).
Impact on family life & friendships	➢ Children must not be separated from parents against their will unless it is in their best interests (9).	➢ Children with disabilities have equal rights to family life and to prevent concealment, abandonment, neglect and segregation (23)
Inadequate health protection & health support	➢ Governments must provide good quality health care & education on health & wellbeing (22).	➢ Right to highest attainable standard of health without discrimination (25).
Inadequate health information on the causes & effects of albinism	➢ Right to reliable information from a range of sources (17).	
Lack of access to justice & few convictions of perpetrators of violence		➢ People with disabilities should have effective access to justice (13).
Lack of voice on issues affecting children with albinism	➢ Right to express their views, feelings & wishes in all matters affecting them & to have their views considered & taken seriously (12).	➢ Right to express their views on all matters affecting them (7). ➢ Right to express themselves & access information (21).

### ‘Barriers to doing’

Evidence suggests that PWA in parts of Africa face many ‘barriers to doing’, structural and physical barriers that prevent them from being included fully in society or having the same opportunities as their non-disabled peers. This is enacted at a personal level (the right to a fulfilling life), at a relational level and at a social level (the right to education).

### The right to live independently

The right to live independently may also be expressed as the right to live a fulfilling life and in the African context, this involves earning a living and supporting the family. Unpublished and anecdotal accounts attest to the challenges faced by PWA as they transition from childhood into adulthood and the potential barriers that this group might face as young adults. Clearly, the impact of childhood experiences has the potential to affect this transition and there is clear evidence that discrimination continues into adult life. Lund’s study of adults with albinism in Zimbabwe highlighted the difficulties encountered in obtaining employment [27]. Baker and colleagues stress the prejudices people with albinism face from employers, emphasising the importance of employment for this group, both economically and for social acceptance [30]. Kiprono et al.’s study on quality of life for PWA in Tanzania recorded that half of his sample (*n* = 128) were unemployed and half of these had experienced discrimination by employers [35]. Those who were employed reported facing many challenges in their work, including sun exposure and vision needs, with 10% describing discrimination in the work place.

### The right to develop relationships

Some studies have accentuated the difficulties some PWA face in developing relationships because of discrimination and stigma [28], especially the importance of acceptance by a partner’s family and friends [27]. The social isolation of children with albinism can be confounded by the barriers they face to being able to play outside with other children and to take part in outdoor activities at school whilst remaining safe from the damaging effects of the sun. As an illustration of prejudice, one study of students in a South African university reported that six out of 10 students who did not have albinism stated that they would not date a person with albinism [36]. In Kiprono et al.’s Tanzanian study, of people who were eligible for marriage over half were single and of those who were married, half reported problems with their partners because of their albinism [35]. The colour of their skin was also cited as a significant cause of divorce or separation. It should be noted that being unmarried is more unusual and stigmatising in many African countries than it is in the global north.

### The right to education

There are mixed policies on the education of children with albinism in Africa, meaning children experience a range of educational experiences. Sometimes they are educated in specialist schools for visually impaired children, although there is an increasing move to adopt inclusive education [12]. However, in some families and communities, children with albinism are not seen as ‘worth educating’ [37] as they are not considered able to contribute in society in the same way as others. An inclusive education study identified gender inequalities in the education of children with albinism in Malawi [37]. Despite albinism affecting boys and girls equally, twice as many boys as girls were attending Resource Centres offering specialist support for their low vision, indicating a gender bias in accessing this service. This study also revealed how parents feared sending their child to school, concerned for their safety while walking to school and lack confidence in teachers’ abilities to keep them safe.

Children with albinism have a right to education, thus attention needs to be given to ensuring that their needs are met within mainstream settings. This can sometimes mean only minor adaptions to classroom layouts, such as access to visual aids, but perhaps more fundamentally requires a change in attitudes amongst teaching staff and other pupils. A study at a special school for children with albinism in rural South Africa identified that although the school had access to magnifiers and low vision devices, these were only used in specific lessons such as map reading [7]. In Zimbabwe and parts of Zambia children with albinism attend mainstream schools where inclusion can be challenging. Teachers can fear teaching a child with albinism [38] and a lack of education and correct information about the condition in the local community inevitably increases the probability of teachers drawing on local myths in their approach to children with albinism [30].

Access to appropriate educational support, including teachers with the knowledge of how to assist children with albinism has been recognized as important in enhancing the self-esteem of these children, promoting their personal development and growth and creating a sense of belonging [1].

Both children’s rights and those of disabled people are compromised by the ‘barriers to doing’ faced by children and adults with albinism, summarised in Table 2.

**Table 2:** Barriers to Doing & the Unenacted Rights of People with Albinism (relevant article in parentheses)

*Barriers To Doing*	*Rights afforded under the UNCRC (1989)*	*Rights afforded under the UNCRPD (2006)*
Reduced educational opportunities due to fear & discrimination	➢ Right to an education (28).	➢ Right to an inclusive education without discrimination (24).
Lack of employment opportunities & discrimination by employers		➢ Right to work, including the right to work in an environment that is inclusive & accessible (27).
Living an independent, fulfilling life	➢ Right to live full & decent lives with dignity & as far as possible, independence & to play an active role in their community (23).	➢ Right to take part in cultural life, recreation, leisure & sport (30).
Segregation from community	➢ Right to relax, play & take part in cultural activities (31).	➢ Countries must make appropriate measures to enable people with disabilities to develop, attain & maintain maximum ability, independence & participation through provision of services (26).
Discrimination in developing relationships/friendships		➢ Right to marry & to found a family.
Lack of voice & empowerment	➢ Governments must actively work to make sure children & adults know about their rights (42).	➢ Governments must raise awareness of the rights of people with disabilities (8).

### A conflict of rights?

As discussed so far, evidence has demonstrated that PWA in Africa face significant barriers both to being and to doing (see Tables 1 and 2), but there is less evidence on how these barriers should be overcome. Within a rights framework it can be argued that many of the rights of the child are not being enacted. Burke et al. [24] contend that the government response in Tanzania to the killing of people with albinism, including increased segregation of children with albinism, has resulted in trade-offs between competing rights, potentially causing further harm to victims and families. PWA face real complexity when the right to life and protection can simultaneously lead to loss of other freedoms and rights, such as a family life, engagement in the community or freedom of movement.

It has been argued that the concept of vulnerability is at odds with a rights framework for PWA [39]. For example, the notion of vulnerability can leverage resources and action to promote protection and ensure PWA’s right to social justice. On the other hand it can also be used to justify social control, which has at times resulted in the placing of children with albinism in secure schools and other institutions. Burke et al. [24] argue that while residential care may sometimes be a life-saving intervention, it cannot be a sustainable solution due to the negative consequences on families and on the social and emotional development of children. Unless interventions are based on a holistic conception of human rights as indivisible, children with albinism will remain socially excluded and segregated from their families and communities [24]. The UNCRPD [13] contains specific obligations on governments to challenge the stereotypes and prejudices experienced by PWA, with what seems to be a patchy response. Segregation, for whatever reason, contravenes fundamental rights to leading independent, fulfilling lives, accessing education and work and developing relationships within local communities. In addition, the voices of children with albinism on this subject have not been heard, despite their right to express their views and choices on matters affecting them. Whilst there is an argument that this might apply to most African children, those with albinism are afforded no voice at all at this time. Clearly significant challenges remain in protecting and promoting the rights of PWA. The evidence suggests that a starting point has to be in ensuring the identification, conviction and punishment of perpetrators of violence towards this group of people and in addressing discrimination and prejudice. Further work is also needed to promote awareness and understanding of albinism and to secure the multitude of other rights being denied to PWA and their families.

### Empowering children and young people with albinism

A qualitative study with 15 participants (aged 18–48 years) in South Africa illustrated that people with albinism want to eradicate myths and misconceptions, lobby for their rights and be treated with dignity and equality [1]. The study suggested that the role of albinism advocacy groups was valued by those accessing them. In particular, the provision of services such as counselling and raising awareness had enhanced self-esteem and a sense of empowerment. Advocacy was also seen as crucial in influencing the media, NGOs and governments. Employment was perhaps not surprisingly also found to be associated with higher levels of self-esteem [1]. Empowering children and young people and advocating for their rights requires financial investment and political will from those in local and national positions of power. Accessing complaints, investigating and reporting mechanisms under the African Children’s Charter and the CRC could be, and has to some extent been, utilised as a potential avenue by advocacy groups to pressure governments and organisations to take human rights violations seriously and improve the human rights of children with albinism. If national legal systems do not provide a remedy for human rights violations, alternatives such as the Optional Protocol to the Convention on the Rights of the Child on a communications procedure (OP3 CRC) which came into force on 14 April 2014 [40], could also provide children themselves with the opportunity to access justice at an international level. However, children will only be able to use this complaints procedure if their States ratify OP3 CRC, and this is yet to happen across most of Africa.

The requirement for countries to fulfil their obligations under the UNCRC and UNCRPD should be a stimulus for action, enacted in legislative measures, implementation plans and full evaluation and measurement of impact of actions [17]. Despite such obligations, politically active disabled people call repeatedly for the strengthening of human rights frameworks which recognise and address the barriers they face [41].

## Conclusion

There is a severe lack of rigorous empirical research into understanding the lives of PWA, especially from their perspective and within a social-relational model of disability. Their voice concerning how their rights are being enacted or denied, the barriers they face and what might better support, empower and protect them has to date been denied. The limited available evidence on living with albinism has been predominately adult focused, with little attention being given to the unique experiences of children. Exploring what is known about children with albinism within an integrated disability and rights lens helps to identify and frame the barriers faced by this group and suggest possible ways to overcome them. The CRC and CRPD together provide a framework for children with albinism and their advocates to call governments and others accountable when their rights are not enacted and thus create, or permit the continuation of, barriers to ‘being’ and ‘doing’. Together these conventions should provide multiple avenues to facilitate the protection and rights of children with albinism.

In addition, our debate has illuminated the need to develop and enact a holistic concept of rights for PWA, which sees human rights as indivisible. Although little attention has been given to the issue, the evidence highlights that in trying to meet the fundamentally critical right to life and protection, other important rights are being denied. This debate has emphasized specific ways in which the lives of PWA could be improved by addressing the ‘barriers to being’ and ‘barriers to doing’. At its heart this requires both a shift in attitude and action to address discrimination among this group of people.

## Abbreviations

NGO: Non-governmental organization
PWA: People with albinism
UNCRC: United Nations Convention on the Rights of the Child
UNCRPD: United Nations Convention on the Rights of Persons with Disabilities
WHO: World Health Organization
